# The tip of the iceberg: postpartum suicidality in Israel

**DOI:** 10.1186/s13584-018-0228-x

**Published:** 2018-06-25

**Authors:** Saralee Glasser, Daphna Levinson, Ethel-Sherry Gordon, Tali Braun, Ziona Haklai, Nehama Goldberger

**Affiliations:** 1 Women & Children’s Health Research Unit, Gertner Institute for Epidemiology & Health Policy Research, Ltd. Sheba Medical Center, Tel Hashomer, 52621 Ramat Gan, Israel; 2 Mental Health Division, Ministry of Health, Jerusalem, Israel; 3 Health Information Division, Ministry of Health, Jerusalem, Israel; 4 Israel Center for Disease Control, Ramat Gan, Israel

**Keywords:** Postpartum, Suicidal ideation, Attempt, Suicide, Israel

## Abstract

**Background:**

Postpartum suicidality, a result of extreme distress or depression, is a tragedy for the woman, infant, and family. Screening for postpartum depression (PPD) is mandatory in Israel, including a question on suicidal ideation. This study presents and analyzes data regarding rates, trends and characteristics of postpartum women who considered, attempted, or completed suicide, to help direct services aimed at preventing these occurrences.

**Methods:**

Suicidal ideation data based on PPD screening was drawn from various publications and databases. Suicide attempt data was obtained from the Emergency Department database for 2006–2015 and matched with the National Birth Registry. Cause of death from the national database for those years were similarly linked to births to identify postpartum suicides and deaths. Postpartum and non-postpartum suicide attempt rates were computed by year, and by age and ethnic/immigrant group. A multivariate logistic model was used to estimate relative risk for postpartum attempts, controlling for age and ethnic group.

**Results:**

Suicidal ideation in recent years has been reported as 1% or less, with higher rates found in studies of Arab women. Suicide attempt rates for non-postpartum women were 3–5 times that of postpartum women, rising over the years, while remaining relatively stable for postpartum women. Adjusted risk of suicide attempt for non-postpartum women was significantly higher; adjusted odds ratio was 4.08 (95% CI 3.75–4.44). It was also significantly higher for Arabs and immigrants from the Former Soviet Union, compared to Israeli-born Jews/veteran immigrants, and for younger women compared to those aged 35–44. Seven postpartum suicides were recorded during 2006–2015, a rate of 0.43 per 100,000 births.

**Conclusion:**

Postpartum suicidality in Israel is low relative to other countries. Although relatively rare and lower than among non-postpartum women, health professionals should be attentive to risk factors, such as past psychiatric disorders, suicide attempts and current emotional distress, particularly among higher-risk populations. The universal screening program for PPD is a valuable opportunity for this, but increased resources should be allotted to implement and utilize it optimally. Prenatal screening should be added as an Israeli Quality Indicator, and postpartum completed suicides should be thoroughly investigated to guide prevention efforts.

*"Y" and her husband were both professionals, financially comfortable, with four children aged seven, five, two, and nine months. After the last birth Y seemed very depressed, "not herself." She didn’t admit to depression, but always had a reason for her mood. For example, if she bought a dress and it didn’t look just the way she thought it would, she became distraught, but her friends told her that her level of distress was not proportional to the situation.*

*Her husband took her to speak to a psychiatrist, but when medication was recommended she absolutely refused. At one point, he turned to the district psychiatrist requesting that she be hospitalized, but the request was denied because he saw no sign that she posed a danger to herself or to her children; she wasn’t hallucinating, and always had an explanation for her behavior.*

*One morning the younger ones were already out of the house and the older girl was getting ready for school, when Y stormed out in a very emotional state, not taking her purse, not saying anything--just got into the car and drove off. The child was scared and called her father, and he came home immediately. They started looking for Y and finally found her near where she had jumped to her death. Afterwards, her family thought that the suicide of Y's good friend following childbirth some years earlier may have influenced her act.* (Note: Details have been changed to protect confidentiality.)

## Background

Suicidality encompasses the spectrum from suicidal ideation (thoughts of self-harm) to suicide attempts, to actual suicide. While these phenomena are relatively rare, they are generally the extreme expressions of distress or depression. Thus, suicidality can be viewed as the ‘tip of the iceberg’ of a broader issue. Depression in general, and postpartum depression (PPD) in particular, have been recognized as significant public health issues [1], and women suffering PPD are at heightened risk for suicidality [2–4]. Diagnosed depression or positive depression screening results have predicted suicidality among postpartum women even when adjusting for other potential risk factors [5, 6], with reports that one in five women screening positive for PPD expressed thoughts of self-harm [3, 7]. PPD itself has serious negative consequences for the woman, the infant and the family [8–10], and suicide attempts and deaths at this time compound the tragedy for the infant and the family.

From a global perspective, rates of postpartum suicide are difficult to glean from the research literature, due to differences in time periods included, the nature of study cohorts, reporting methods, and years under consideration. Thus rates of postpartum suicide per 100,000 live births have varied. For example, in Washington State [11] it was reported at 1.4, in Finland [12] at 5.9, while in Taiwan it was 6.9 [13]. Many studies related to the entire perinatal period [i.e. pregnancy and the year following childbirth], including reports of perinatal suicide rates per 100,000 live births of 2.6 in Canada [14], 2.0 in the UK [15], and 3.7 in Sweden [16]. The U.S. National Violent Death Reporting System [17] reported a rate of 2.0, while the rate in Colorado was reported as 4.6 [18]. Reports from several countries of varying income levels have found that suicide is among the main causes of maternal mortality in the year following childbirth [1, 8, 14, 15, 19, 20]. It has been noted both in UK and Australia that the reduction in maternal mortality rates in recent years have not been paralleled by a decrease in the rate of maternal deaths by suicide [21, 22].

Of the very few reports of postpartum suicide attempt rates, Weng [13] found a rate of 9.9/100,000 live births in Taiwan, and Schiff [11] reported a much higher rate of about 43.9/100,000 live births in Washington State over a 15-year period. Compounding the risk, it has been repeatedly reported that the methods used during the postpartum period are distinct in their violent nature [hanging, jumping from height] compared to female suicides at other periods of life [14–16, 19, 20], implying high intent, thus attempts are more likely to result in death.

Many factors associated with postpartum suicide risk may be modifiable, and therefore deserve attention as a public health priority [1, 7]. The rate of suicide attempts in the postpartum year can reflect the scope of severe depression, and suicidal ideation is an obvious risk factor for subsequent suicide attempt and completion [23], thus being alert to such thoughts may enable identification and intervention with women at risk. Although the tip of this iceberg--completed suicide--is a rare event, its devastating result warrants attention to this outcome, as well as to those which are likely to presage it--suicide attempts and suicidal ideation.

In light of the fact that women in the perinatal period are usually monitored by health professionals, the Israel Ministry of Health [MOH] has recognized the opportunity for early identification of this problem, and a MOH Directive was issued mandating a program for early identification of women at risk or suffering from PPD [24, 25]. The program is implemented by public health nurses in all Mother-and-Child Healthcare Centers [locally referred to as *Tipot Chalav*, i.e. “Drop of Milk”]. These clinics are universally available for pregnancy follow-up and provision of all vaccination and well-child visits from birth to 6 years old. The PPD identification program includes three elements: universal screening using the Edinburgh Postnatal Depression Scale [26], followed by nurses’ non-directive, supportive counselling intervention, and referral to mental health services for diagnosis and treatment as necessary.

While the scope of the problem worldwide is cause for concern, little information regarding the rates of postpartum suicidality in Israel has been reported to date. In general, although data regarding suicide is universally recognized as an underestimate, due to factors related to determining the circumstance of death and financial considerations [27], in Israeli society there is further stigma stemming from religious beliefs prohibiting self-harm, as well as the cultural norm regarding military combat casualties at the top of the “death hierarchy” and suicide victims at the bottom [28, 29]. These often result in labeling suicides as “undetermined,” “accidental,” or “other” causes of death.

The objective of the current study is to present and analyze the available data regarding rates, trends and characteristics of those who might consider, have attempted, or committed suicide, and thus hopefully direct services aimed at preventing these occurrences.

## Method

Two methods of data gathering were used in this study. The information regarding suicidal ideation was compiled by reviewing and summarizing various published and unpublished data on postpartum women only. The data regarding suicide attempt and completed suicide rates is the result of new analyses conducted for the current study comparing postpartum and control groups of women.

### Suicidal ideation

Reported here are data from various sources that offer an estimation of the rate of suicidal ideation among Israeli women in the postpartum period (Table 1) [30–35]. The most current and broad data, albeit as yet unpublished, were communicated from the MOH Tipat Chalav database (Rubin, L., personal communication, 2017) and Maccabi Health Services database (Fish, R., personal communication, 2017). All data is based on responses to the Edinburgh Postnatal Depression Scale [26, 36] (EPDS), the most widely used instrument in research dealing with perinatal mental health. The EPDS is a 10-item screening instrument that includes a question regarding thoughts of self-harm (Question 10) that states: “In the past week the thought of harming myself has occurred to me: quite often/sometimes/hardly ever/never.” Any response other than ‘never’ is considered suicidal ideation, and guidelines instruct immediate consultation in the event of this response [25]. After its successful use in Israel in a pilot project in MOH Mother-Child Healthcare clinics [30], its implementation for universal screening of depressive symptoms during pregnancy and within the first postpartum months was gradually expanded, and since 2013 has been mandatory.

**Table 1:** Rate of postpartum women responding positively to Question 10 on the Edinburgh Postnatal Depression Scale

Source	Year^a^	N	Region/area	Q10 > 0 (%)	Comment
Glasser et al. [34]^b^	1995	288	Central Israel	8.7	Low SES Jewish population, high rate of new immigrants
Glasser et al. [30]^b^	2001	259	Central & Southern Israel	0.8	Five cities
Glasser [35]	2003–5	6612	Country-wide	0.9	Six HMO districts (range 0.7–4.1%)
Glasser et al. [31]	2009	2764	Northern Israel	2.1	Arab population
Alfayumi-Zeadna et al. [32]	2008–9	564	Southern Israel	2.7	Beduin population
Cwikel et al. [33]	2014	1000	Central Israel	1.0	Jewish population
Rubin^c^	2015	131,905	Country-wide	0.4	Ministry of Health database^d^
Fish^e^	2014–6	22,423	Country-wide	0.3	HMO database, generally of higher SES

^a^Year of data collection

^b^Data not included in publication

^c^Rubin, L., Ministry of Health, personal communication, 2017

^d^Sample not necessarily representative, but based on broad geographical distribution

^e^Fish, R., Maccabi Health Services, personal communication, 2017

### Suicide attempts

Data on non-fatal suicide attempts were obtained from the National Hospital Emergency Department [ED] database, which is maintained by the MOH. This includes all ED admissions, with demographics, reason for admission, diagnosis, date and time of admission and discharge, and ED discharge destination. Individuals’ identity numbers are encrypted to protect patient privacy, but allow matching of records belonging to the same individual. The data were cross-checked by encrypted identity number with the National Birth Registry, to identify women who were admitted to ED for a suicide attempt within one year of delivery.

The present analysis included individuals admitted to the ED in all general hospitals in Israel during 2006–2015 whose reason for admission was classified as attempted suicide and/or had an ED diagnosis of suicide (ICD-9 codes E950-E959), and were aged 18 to 44 years-of-age. Psychiatric hospitals and those in East Jerusalem are not included in this database.

Suicide attempts were characterized by age group (18–24; 25–34; and 35–44) and ethnic group. As of 2016 the population of Israel was comprised of 74.8% Jews, 20.8% Arabs, and a small percentage (4.4%) of “Others” [non-Jewish Israelis who are not Arabs, many of whom are immigrants or relatives of immigrants from the Former Soviet Union (FSU)]. For this analysis, the data presented for Jews include “Jews and Others.” The group of Jews was further divided into four sub-groups: Israeli-born or veteran immigrants (before 1990), and recent immigrants (since 1990) from either the FSU, Ethiopia, or from other countries. The postpartum period was defined as one year following childbirth. The control group included all other suicide attempts recorded in the ED database for women in this age group.

The population denominator used to calculate rates in the postpartum group was taken as the mid-year population of women after birth for each year and group, estimated from the National Birth Registry as those within a year of birth on June 30th of each year. The control group populations were calculated as the difference between the postpartum population and mid-year total population estimates provided by the Israeli Central Bureau of Statistics (CBS) for each age and ethnic/immigrant group.

Suicide attempt rates and rate ratios with 95% confidence intervals (CI) were calculated for the postpartum and control group by year, ethnic group, age, and immigrant status group for the study period. To assess the effect of all characteristics together, a multivariate logistic model was built predicting suicide attempt by postpartum status, age group and ethnic/immigrant group.

#### Measure of severity of attempts

A measure of severity of suicide attempts was assessed for the postpartum and control groups by calculating the number of suicides as a percentage of total suicides plus non-fatal suicide attempts, for the period 2006–2015.

### Suicides

Data on completed suicides of women aged 18–44 were taken from the national database of causes of death, maintained by the CBS, based on death certificates. Since 1998, the CBS has coded the underlying cause of death according to the International Classification of Diseases Version 10 [ICD-10]. This data was cross-checked with the National Birth Registry to identify cases of maternal death within one year of delivery, for the period 2006–2015.

### Analysis

Suicide and suicide attempt data were linked and analyzed with SAS 9.4 software (Cary, NC: SAS Institute Inc.).

### Ethics

The study was approved by the IRB committee of the Israel Ministry of Health (MOH 029–2017).

## Results

### Suicidal ideation

Several studies in Israel have reported response rates to Question 10 on the EPDS during the postpartum period (Table 1). The rate of positive responses has ranged from 8.7% in an early study in a low socioeconomic area with a large proportion of new immigrants, to 1% or less in more recent studies of the general Israeli population. Higher rates, of 2–3%, have been reported in studies of Israeli Arab women. A study conducted by the Clalit Health Fund [36] (not shown), enabled analysis of the data by geographical region and also by type of community (urban, rural, etc.). In that cohort the rates of suicidal ideation ranged from 0.8 to 3.3% among the regions and from 0.8 to 5.7% among the different types of community.

### Suicide attempts

**Fig. 1:**
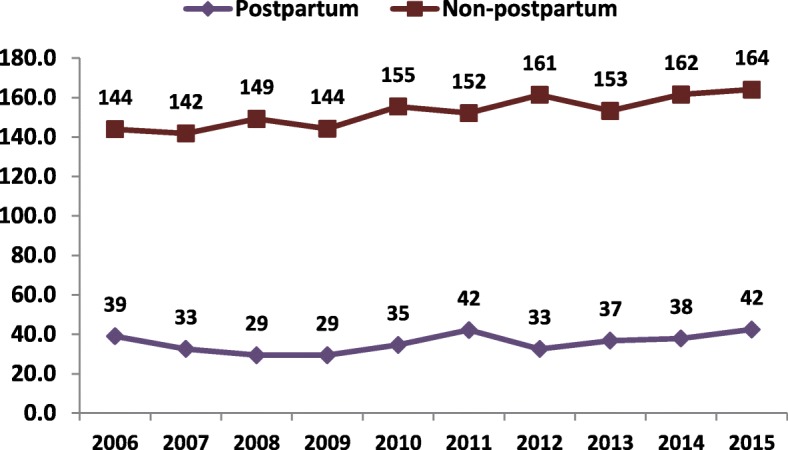
Suicide attempt rates of women aged 18–44 within one year postpartum compared to non-postpartum women, by year: 2006–2015; rate/100,000 persons

During 2006–2015, 20,259 cases of suicide attempts by women aged 18–44 were recorded attending the EDs of general hospitals in Israel. Of these attempts, 565 (2.8%) were by women in the postpartum period. Fig. 1 presents the suicide attempt rate for postpartum compared to non-postpartum women by year. The suicide attempt rate for non-postpartum women was three- to five-times that of postpartum women. The rate among non-postpartum women, 144 per 100,000 population in 2006, was relatively stable until 2009 and then increased, reaching 164 in 2015. For postpartum women, the rate varied between a peak of 42 per 100,000 in 2011 and 2015, with the lowest rate of 29 in 2009.

The suicide attempt rate for the total period 2006–2015 was 36 per 100,000 population for postpartum women, and for non-postpartum women it was 153, i.e., 4.3 times greater. Suicide attempt rates for both postpartum and non-postpartum women decreased with age, and the rate ratio non-postpartum/postpartum increased (Table 2). Rates were lower for Jews in both groups compared to Arabs, and the rate ratio non-postpartum/postpartum was higher for Jews, 5.7 (95% CI 5.1–6.4) compared to 2.7 (95% CI 2.4–3.1) for Arabs.

**Table 2:** Population characteristics of suicide attempts by postpartum and non-postpartum women, aged 18–44, 2006–2015

		Postpartum	Non-postpartum	Postpartum	Non-postpartum	Rate ratio non- postpartum/ postpartum
		Number		Rate/100,000 population		
Total		565	19,694	35.8 (32.9–38.9)	153.0 (150.9–155.2)	4.3 (3.9–4.7)
Age Group	18–24	214	9359	71.8 (62.5–82.1)	250.0 (244.9–255.1)	3.5 (3.0–4.0)
	25–34	278	6104	29.6 (26.3–33.3)	131.5 (128.2–134.8)	4.4 (3.9–5.0)
	35–44	73	4231	21.3 (16.7–26.8)	94.3 (91.5–97.2)	4.4 (3.5–5.6)
Ethnic Group^a^	Jews^b^	301	14,621	25.2 (22.4–28.2)	143.1 (140.8–145.5)	5.7 (5.1–6.4)
	Arabs	263	4972	68.5 (60.4–77.2)	187.3 (182.1–192.6)	2.7 (2.4–3.1)
Immigrant status (for Jews^2^)						
FSU immigrants (recent^c^)		64	2871	58.8 (45.3–75.1)	171.9 (165.7–178.3)	2.9 (2.3–3.8)
Ethiopian immigrants (recent^c^)		13	231	91.5 (48.7–156.5)	137.6 (120.4–156.5)	1.5 (0.8–2.7)
Other immigrants^d^ (recent^c^)		10	558	20.9 (10.0–38.4)	145.1 (133.3–157.6)	7.0 (3.5–13.8)
Israeli-born/veteran immigrants		214	10,961	20.9 (18.2–23.9)	138.0 (135.4–140.6)	6.6 (5.7–7.6)

^a^Not including unknown. Ethnicity unknown for 102 attempts

^b^Jews includes other non-Jewish Israelis who are not Arab (4.4% of the total population)

^c^Since 1990

^d^Not including FSU and Ethiopian immigrants

Among Jewish postpartum women, immigrants from the FSU and Ethiopia had the highest suicide attempt rates, while for non-postpartum women FSU immigrants had higher rates, but Ethiopians did not. Hence the rate ratio non-postpartum/postpartum was lowest for Ethiopians, 1.5 (95% CI 0.8–2.7) compared to 2.9 (95% CI 2.3–3.8) for FSU immigrants and 6.6 (95% CI 5.7–7.6) for Israeli-born or veteran immigrants, respectively.

**Fig. 2:**
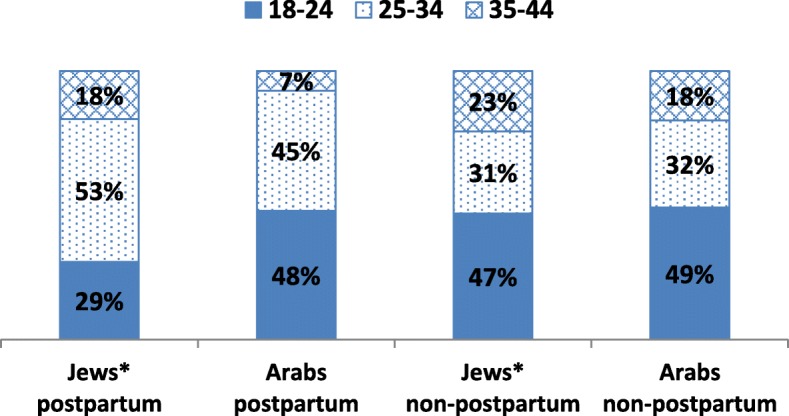
Suicide attempts among postpartum and non-postpartum women during 2005–2016: age distribution among Jews and Arabs

Comparison of the suicide attempt age distribution for Jews with that of Arabs in the postpartum and non-postpartum groups (Fig. 2), shows that for non-postpartum women it was very similar for both the Jewish and Arab women, with about half of the attempts in the 18–24 age group and about 30% in the 25–34 age group. However among postpartum women, the distribution differed; while for Arab women almost half of the attempts (48%) were in the 18–24 age group and only 7% in the 35–44 age group, the corresponding proportions for Jewish women were 29 and 18%.

Multivariate logistic regression analysis predicting the risk for suicide attempt (Table 3) showed similar results to those reported above. Non-postpartum women had a fourfold higher risk of suicide attempt than did those within a year postpartum; when controlling for age and ethnic/immigrant group, the adjusted odds ratio (AOR) was 4.08 (95% CI 3.75–4.44). Arabs had a significantly higher suicide attempt risk compared to Israeli born/veteran immigrants (AOR = 1.37, 95% CI 1.32–1.41), even when controlling for age and postpartum status, as did immigrants from the FSU (AOR = 1.39, 95% CI 1.33–1.44). However, Ethiopian immigrants did not have a significantly different risk when controlling for the other factors. The risk of suicide attempt in the youngest age group, 18–24, was over two-and-a-half times that of the oldest group, aged 35–44 (AOR = 2.67, 95% CI 2.58–2.77), while women aged 25–44 also had an elevated risk.

**Table 3:** Multiple regression analysis to predict suicide attempt, 2006–2015

Effect	Odds ratio (95% CI)
Whether after birth: Non postpartum vs. postpartum	4.08** (3.75–4.44)
Ethnic/immigrant group:	
Arabs vs. Israeli-born/veteran immigrants	1.37** (1.32–1.41)
Ethiopia vs. Israeli-born/ veteran immigrants	0.99 (0.87–1.12)
FSU vs. Israeli-born/veteran immigrants	1.39** (1.33–1.44)
Other immigrants vs Israeli-brn/veteran immigrants	1.04 (0.96–1.13)
Age:	
18–24 vs. 35–44	2.67** (2.58–2.77)
25–34 vs. 35–44	1.39** (1.34–1.45)
C statistic	0.644
N of events	20,157

**P-value: < 0.001

### Suicide

During the decade 2006–2015 there were 193 deaths among women aged 18–44 within the year following childbirth. Causes of death as recorded by the CBS are presented in Table 4.

**Table 4:** Causes of death in the first postpartum year, 2006–2015

ICD-10 Code	Cause	Number	Percentage of deaths
	Natural causes - total	155	80.3
O00-O99	Obstetric death	61	31.6
C00-C97	Cancer	40	20.7
R96-R99	Unknown/ill-defined	18	9.3
I00-I09,I11,I13,I20-I51	Heart disease	5	2.6
I60-I69	Cerebrovascular disease	4	2.1
J00-J99	Respiratory	4	2.1
B20-B24	HIV	2	1.0
Remaining natural causes	Other natural	21	10.9
	External causes - total	38	19.7
V01-V99, Y85	Accidental transport deaths	14	7.3
X85-Y09, Y87.1	Assault	9	4.7
X60-X84, Y87.0	Suicide	7	3.6
Y37	War and terror	3	1.6
W00-W19	Accidental falls	2	1.0
Y10-Y34	Undetermined intent	2	1.0
W20-X59, Y86	Other accidents	1	0.5

About one-fifth of the deaths (38/193) were due to external causes, only seven of which were registered as intentional self-harm (suicide), comprising 3.6% of the postpartum deaths. The rate of reported suicide per 100,000 live births was 0.43. It was noted that three of these seven suicides were of Arab women.

It is likely that the true number of suicides is somewhat higher. For example, some of those with external causes classified as accidental fall, other accidents, or of undetermined intent may actually have been suicides, as well as some of those with unknown or undetermined cause of death.

#### Suicides as a proportion of suicidality

The proportion of suicides from the total number of suicide attempts plus suicides among postpartum women aged 18–44 during 2006–2015 was 1.2%, while the corresponding rate for non-postpartum women was 0.4% (84 completed suicides).

## Discussion

The present study reports Israeli data regarding the rates of suicidal ideation, suicide attempts, and completed suicides among women in the year following childbirth.

### Suicidal ideation

From the available Israeli published reports [30–35] and the as-yet unpublished data from the MOH and Maccabi Health Services databases, the rates of postpartum suicidal ideation, as reflected by any score other than zero on Question 10 of the EPDS screening questionnaire (thoughts-of-self-harm), are considerably lower than those reported in other countries. In a study of over 1000 women in New York, Bodnar-Deran [37] found that 6% of the participants presented with suicidal ideation during the first six months postpartum. Among 1500 pregnant women in Peru, 8.8% of the participants responded in the affirmative to Question 10 [38]. Howard [39] reported that by 18 weeks postpartum 9% of the 4150 women who completed the EPDS reported some suicidal ideation (including hardly ever); 4% reported that the thought of self-harm themselves had occurred sometimes or quite often. In that study, multivariate analysis indicated that suicidal ideation was associated with younger age, higher parity and higher levels of depressive symptoms, and endorsement of ‘yes, quite often’ to Question 10 was associated with affirming at least two clinical interview items on suicidality. In Lindahl et al.’s extensive review of this topic [19], postpartum suicidal ideation rates ranged from 4.6 to 15.4% in countries including the United States, Canada, Britain, Finland, South Africa and India.

In Israel, the only exception to the low rates is the study reporting data collected in 1995 [34], in a low socio-economic community with a large proportion of new immigrants. That rate, 8.7%, is somewhat higher than the 6.2% of suicidal ideation reported by adult females in the Israel National Health Survey, conducted in 2003–2004 [40]. The study was conducted before 2001, when the first systematic effort in Israel by the MOH was begun, raising awareness among primary care nurses and the public regarding PPD and its early detection [24, 30]. In the ensuing years, even before 2013 when the MOH mandated EPDS screening [25], attention was increasingly paid to early identification, screening and supportive intervention of maternal emotional distress by public health nurses during pregnancy and in the first two months postpartum [31, 35]. Interestingly, a U.S. study of trends in PPD symptoms [41] found an overall decline from 14.8% in 2004 to 9.8% in 2012 among thirteen states that had data over this period. It is possible that the increasing awareness and intervention, in some cases beginning during pregnancy, has contributed to the lower rates of postpartum suicidal ideation reported in more recent years, as seen in Table 1.

### Suicide attempts

The rate of suicide attempts was considerably lower among postpartum women compared to non-postpartum women for all years and in all groups considered in this analysis. This was also the conclusion of Lindahl et al.’s review of 27 studies [19]. The relatively stable rate among Israeli postpartum women between 2006 and 2015, with some years having lower rates, may also reflect the impact of increasing awareness due to the universal screening program, particularly since the rate among non-postpartum women increased considerably over this period.

The overall rate of postpartum suicide attempts in Israel between 2006 and 2015 was 35.8 per 100,000 population, lower than that reported by Schiff et al. [11] of 43.9 per 100,000 live births in Washington State. The difference is actually greater, since Schiff et al. reported only hospitalized suicide attempts, while this study included all ED-admission attempts, of which only 43% were hospitalized in the postpartum period. In Taiwan, Weng et al. [13] reported even lower rates of 9.9 per 100,000 live births, but they also appear to have identified only women admitted to hospital following ED admission for serious suicide attempts, since they found only 139 attempts in 2002–2012, very few compared to the 95 completed suicides.

Some groups were found in this study to be at a higher risk than others. For example, risk was highest for mothers in the youngest age group, similar to the results of Schiff et al.’s [11], and Gressier et al.’s [5] finding that among women hospitalized in psychiatric Mother-Baby Units, younger age was a risk factor for postpartum suicide attempt. Arab postpartum women were at a higher risk, with lower rate ratios compared to non-postpartum women. Contributing to this was the higher proportion of young suicide attempters among the postpartum Arab women compared to Jewish women. One factor might be the inequalities in health service utilization between the Jewish and Arab sectors, with less utilization of specialist and mental health services among Arab women [42, 43]. This also may reflect the younger median age at which Arab women give birth, which has remained stable over the past decade, compared to that of Jewish women, which has been rising [44]. Even the age-adjusted odds ratio showed a 37% higher suicide attempt risk for Arab women compared to Jewish Israeli-born/veteran immigrants. The age-adjusted risk was also higher for postpartum FSU immigrants, similar to their higher risk for suicide and suicide attempts reported in the general population [45]. Indeed, the higher suicide attempt rates in both groups reflect the heightened stress experienced by these groups; Arab women as members of a disadvantaged minority as well as women’s subordinate position in their traditional, patriarchal community, and FSU immigrants with the stresses of immigration and the high rate of single mothers in this group [46, 47].

In the present study postpartum suicide attempt rates per 100,000 population were calculated, enabling comparison with rates of the non-postpartum population; this is in contrast to other studies that only calculated the rates per 100,000 live births. However, the two rates are very similar. For example, in this study the overall suicide attempt rate was 35.8 per 100,000 population and 34.6 per 100,000 live births.

### Suicide

In several studies suicide has been cited as one of the leading causes of maternal death, especially, but not uniquely, among women suffering depression or with previous psychiatric history [15, 16, 19, 20]. While in Israel suicide was an important cause of death in the first postnatal year, the rate of 0.43 per 100,000 live births, or 3.6% of postpartum mortality, was considerably lower than that found elsewhere. For example, in the Canadian 15-year population-based study [14], the suicide rate among women in the postpartum year was 1.57 per 100,000 live births, which comprised 6% of postpartum mortality. Metz et al. [18] reported a rate of 4.6 per 100,000 live births in Colorado from 2004 to 2012, and Esscher et al. [16] reported 3.7 per 100,000 live births during 1980–2007 in Sweden, which amounted to18% of maternal deaths. Even assuming an underestimation of suicides in Israel of 42%, as found by Bakst et al. [27], the revised rate of 0.61 per 100,000 live births is still low compared to other studies. On the other hand, Fuhr et al.’s [48] meta-analysis of studies from 21 middle- and low-income countries found a pooled prevalence of pregnancy-related or maternal deaths attributed to suicide of 1.0%, lower than that in Israel. However, this may be an underestimate since many of the studies included reported only deaths in the first 42 days postpartum, while suicides have been shown to often occur later in the year following childbirth [21].

The postpartum suicide rate in Israel is low compared to the nationwide suicide rate. For example, the national rate in 2011–2013 for females aged 25–44, was 2.4 per 100,000 population [45], more than five times the postpartum rate. The low postpartum suicide and suicide attempt rates in Israel compared to other high-income countries are consistent with Israel’s low overall suicide rates compared to international data [45].

One factor involved may be the protective effect of religiosity, since a disproportionate number of births in Israel are to religious women, both Arab and Jewish [49–51]. This protective effect of religiosity has been found in various societies [52–54], and in Israel was shown by Glasser et al. [32] who reported lower rates of antenatal depression among Arab women with increasing religiosity, and by Dankner et al. [55] regarding PPD among Jewish women in Israel. Mann [56] reported that increasing religiosity antenatally was associated with lower rates of PPD, and Van Praag [57] has noted the protective effect of religion in suicide prevention. In addition to the general protective effect of religiosity, both Judaism and Islam forbid suicide. On the other hand, this may be a “two-edged sword,” since heightened stigma in religious societies regarding mental health disorders [58–61] can lead to under-reporting of suicide and therefore lower reported, but not actual, rates. Indeed, while Russo et al. [62] found that religion was among the positive influences on their emotional well-being among Afghan women interviewed in Australia, they also noted their cultural stigma associated with mental illness, contributing to resistance to obtaining professional support.

Although in this study the suicide rate was lower among postpartum than non-postpartum women, as also reported by Lindahl et al. [19], postpartum suicides were found to comprise a higher proportion of suicidality than were non-postpartum ones. This may indicate the greater lethality of postpartum suicide attempts, an aspect supported by several reports that note the violent methods employed in postpartum suicides, such as hanging, jumping or falling [15, 16, 19, 20, 63]**.**

Several problems arise universally when attempting to document or monitor postpartum suicide rates. While international comparisons of cause of death are based on the assumption of equivalence of coding practices and definitions, deaths during pregnancy or postpartum are divided into direct, indirect or incidental [20, 64]. Direct deaths are the result of obstetric complications (ICD-10 codes O00-O97); indirect deaths result from aggravation of a condition by pregnancy; and incidental maternal deaths occurred during pregnancy or postpartum, but were not likely caused by it. Maternal mortality statistics include direct and indirect maternal deaths. In 2012, new World Health Organization (WHO) guidelines defined postpartum suicide as a direct cause of maternal mortality thus expanding these cases and leading to increased maternal mortality rates [65].

Definitions also differ with respect to the period under consideration. Unlike the present study, which dealt with the first postpartum year, international data on maternal death often only include deaths within 42 or 90 days of childbirth, such as many in Fuhr et al.’s meta-analysis [48]. Official Israeli data on maternal mortality includes only deaths related to, or aggravated by, pregnancy and up to 42 days postpartum, while the present study reported all deaths in the postpartum year, irrespective of their association with childbirth. Other countries include all pregnancy-related deaths in reported statistics, thus including prenatal suicides [48, 63]. ICD-10 expanded the concept by defining “late maternal deaths” (> 42 days to one year postnatally) [64]. It has been noted, however, that late maternal deaths are less likely to be documented as such [20, 66]. In the UK Confidential Enquiries into Maternal Death, later deaths were found by linking death records with births in the previous year [15]. When suicides from these later deaths, not initially reported, were included as maternal deaths, suicide was the leading cause of maternal death, compared to other direct causes divided into major subgroups. The 2016 MBRRACE-UK Report [21] has similarly concluded that maternal suicide remained the leading cause of direct deaths that occurred during pregnancy or up to one year postpartum. In a review of maternal deaths in Australia, Thornton et al. [66] found that there was a nearly four times likelihood of maternal deaths from external causes in the 9 to 12 months postnatally, compared to the first three months. Thus reporting only early postpartum data would have a direct impact on maternal suicide rates reported, and may contribute to the low prevalence found by Fuhr et al. as noted above [48]. Maternal deaths related to psychiatric illness are also increasingly being included as late maternal deaths [20].

Another problem is that under-reporting of suicide in general [66, 67] and in the postpartum period specifically [20, 68, 69], is well known and may be attributed to misclassification, or stigma in some societies, as noted above. In Fuhr et al.’s review [48], the rate rose from 1.00 to 1.68% when reclassifying the leading suicide methods from injury to suicide. An in-depth investigation (primarily from police reports) of deaths in Israel with a recorded cause that could mask suicide, such as unknown cause or of undetermined intent, indicated that the suicide rate was underestimated by 42% [27]. In Israel particularly, the factors that might support under-reporting include both the heightened stigma in religious societies regarding mental health disorders [58–61] and the Israeli culture of the “death hierarchy,” whereby fallen soldiers are at the pinnacle and suicides at the bottom [28, 29].

### Strengths and limitations

The strength of this study is that the suicide and suicide attempt findings are based on data for the entire Israeli population over a long period, allowing analysis of the suicide attempts by population characteristics, and comparison of postpartum rates with those of the rest of the population. There are some limitations as well. Suicidal ideation data available is primarily descriptive in nature and no in-depth analysis was presented. The relatively new MOH *Tipat Chalav* database could enable such analysis to gain a better understanding of the characteristics of women who express such ideation, and it is hoped that in the future this data will be available so that prevention efforts can be focused on these at-risk women. The suicide attempt data did not include East Jerusalem hospitals. This may lead to under-estimation of Arab suicide attempt rates, which may be still greater than the already higher rates compared to Jews that were reported in this paper, for both postpartum and non-postpartum women. Also, in light of the fact that some of the sub-populations were very small, interpretation of the results should be taken with caution. Data from psychiatric hospital ED’s was also not included in the database used, but since most serious suicide attempts cause physical injury, they are usually referred to general rather than psychiatric hospitals, so this should not greatly affect the results. Regarding completed postpartum suicides, due to the small absolute number recorded, a consequence of the rare nature of this event, no statistical analysis of them could be undertaken by specific socio-demographic characteristics. Access to the psychiatric history of the postpartum women was not available for this study, although in other studies a significant proportion of women who committed suicide in the postnatal period were found to have had psychiatric treatment before or during pregnancy [14, 16, 20]. Although this could not be verified in the present study, an earlier study on suicides in the general Israeli population found a greatly elevated suicide risk among those who had past psychiatric hospitalizations [70]. Thus psychiatric history is clearly a risk factor that should be assessed in contacts with postpartum women. As mentioned above, the reliability of suicide registration is also a limitation.

## Conclusion and policy recommendations

Although postpartum suicidality is relatively rare, awareness of the possibility is important. While the rates of suicide attempts and completed suicides in the postnatal period are lower than those of similarly-aged women who have not given birth in the previous year, following childbirth women are more likely to be repeatedly exposed to primary healthcare providers [e.g., public health nurses, pediatricians] for both their own and their infants’ care. Hence it is important that these professionals be alert to the issue of postpartum suicidality. In Israel, universal screening is conducted among pregnant and postpartum women for signs of depressive episodes and specifically for suicidal ideation. This screening and contact offers an appropriate opportunity for these professionals to be attentive to risk factors, such as past psychiatric disorders, past suicide attempts and signs of extreme current emotional distress [2, 3, 37, 71], and hopefully intervene to prevent the escalation that could lead to suicide attempts or suicide [72]. The data presented here may support the value of this important program in maintaining low levels of postpartum suicidality, however in reality additional resources have not been allotted since mandating the program, placing an increased burden on already overworked staff [30, 35]. Glavin et al. [73] found that public health nurses spent an extra 20 min for initial postpartum screening and discussion, and 30 min for each supportive counselling session with women who expressed depressive symptoms. It is therefore recommended that appropriate resources be allotted to those implementing the program so that they can conduct the screening and intervention in an optimal manner, with particular attention paid to the Arab and FSU immigrant populations. While this may seem intuitive, it is also recommended that advantage be taken of the large databases which have been created by the Ministry of Health and the HMO’s to document and analyze the association between the use of screening and intervention program and actual rates of PPD, suicidal ideation, attempts or completed suicides in Israel.

Since depression during pregnancy has been found to be a significant predictor of subsequent PPD [74–76], early intervention is recommended prior to delivery, which could contribute to prevention of both PPD and postpartum suicidality in all its expressions, as supported by the research of Yazici et al. [77]. The 2007 Confidential Enquiry into Maternal and Child Health noted that identifying and intervening with women at potential risk in the antenatal period, seem to be having a beneficial effect in reducing postpartum suicide [68]. Currently, postpartum EPDS screening is included as one of the Israeli MOH Quality Health Indicators [78], requiring that all Mother-Child Health Care Clinics report implementation of the postpartum screening. This focuses attention on and adherence to conducting the screening following delivery. However, although EPDS screening during pregnancy is also mandated by the Ministry of Health Directive [25], it is not included as a Quality Indicator. It is therefore recommended that the antenatal screening be included as an additional required Quality Health Indicator, in order to raise awareness and encourage compliance with the screening, and intervention as necessary, at the earlier stage, hopefully serving as an important and effective preventive measure.

Statistics--particularly with regard to relatively rare events--only tell part of the story. It is recommended that an audit or psychiatric autopsy [67, 79, 80] be conducted for each case of postpartum suicide, as this would assist in better understanding the precursors of these events in order to enable health professionals to more reliably recognize impending dangers to women with whom they have contact. As Cantewell et al. stated in the 2011 report of the Confidential Enquiries into Maternal Deaths in the UK [81]. “Investigations into deaths from psychiatric causes at any stage during pregnancy and the first postnatal year should be carried out and should be multi-agency, and include all the services involved in caring for the woman” (pg. 41). Similar efforts characterizing perinatal suicide attempts could also offer important information to guide health policy in ensuring close follow-up of such cases and hopefully reducing these tragic occurrences. A multi-pronged effort to intervene early for primary and secondary prevention of antenatal and postnatal depression, and to better understand perinatal suicide attempts and completed suicides, could assist in addressing the base of the suicidality pyramid, and in turn hopefully help to reduce the most tragic events at the tip of the iceberg.

## Abbreviations

AOR: adjusted odds ratio
CBS: Central Bureau of Statistics
CI: confidence interval
ED: Emergency Department
EPDS: Edinburgh Postnatal Depression Scale
FSU: Former Soviet Union
HMO: Health Maintenance Organization
ICD-10: International Classification of Diseases Version 10
MOH: Ministry of Health
PPD: postpartum depression
WHO: World Health Organization
